# Thyroid Function in Korean Adolescents with Obesity: Results from the Korea National Health and Nutrition Examination Survey VI (2013–2015)

**DOI:** 10.1155/2018/6874395

**Published:** 2018-09-03

**Authors:** Won Kyoung Cho, Hyo-Kyoung Nam, Jae Hyun Kim, Young-Jun Rhie, Sochung Chung, Kee-Hyoung Lee, Byung-Kyu Suh

**Affiliations:** ^1^Department of Pediatrics, St. Vincent's Hospital, College of Medicine, The Catholic University of Korea, Suwon, Gyeonggi-do 16247, Republic of Korea; ^2^Department of Pediatrics, College of Medicine, Korea University Guro Hospital, 148 Gurodong-ro, Guro-gu Seoul 08308, Republic of Korea; ^3^Department of Pediatrics, Seoul National University Bundang Hospital, 82 Gumi-ro 173 Beon-gil, Bundang-gu, Seongnam, Gyeonggi-do 13620, Republic of Korea; ^4^Department of Pediatrics, College of Medicine, Korea University Ansan Hospital, 123 Jeokgeum-ro, Danwon-gu, Ansan, Gyeonggi-do 15355, Republic of Korea; ^5^Department of Pediatrics, College of Medicine, Konkuk University School of Medicine, 120-1 Neungdong-ro, Gwangjin-gu, Seoul 05030, Republic of Korea; ^6^Department of Pediatrics, College of Medicine, Korea University Anam Hospital, 73 Inchon-ro, Seongbuk-gu Seoul 02841, Republic of Korea; ^7^Department of Pediatrics, Seoul St. Mary's Hospital, College of Medicine, The Catholic University of Korea, 222 Banpo-daero, Seocho-gu, Seoul 06591, Republic of Korea

## Abstract

**Purpose:**

In this study, we investigated the status of thyroid function and its association with metabolic risk factors in Korean adolescents.

**Methods:**

Among 2679 subjects aged 10–19 years who participated in the Korea National Health and Nutrition Examination Survey VI (2013–2015), 1067 adolescents (*M* = 559, *F* = 508) with available data on free T4 (FT4) and thyroid-stimulating hormone (TSH) were included. Study participants were classified into normal weight [body mass index (BMI) below 85th percentile, 80.7%], overweight (85th ≤ BMI< 95th percentile, 8.7%), and obesity (BMI ≥ 95th percentile, 10.6%).

**Results:**

With increasing levels of BMI category, the means of TSH increased (2.73 ± 0.06, 2.77 ± 0.02, and 3.24 ± 0.22 mIU/L, *P* = 0.031) and FT4 decreased (1.30 ± 0.01, 1.26 ± 0.02, and 1.25 ± 0.02 ng/mL, *P* = 0.001). Positive linear associations were observed between TSH and BMI *z*-score (*P* = 0.031), waist circumference (*P* = 0.013), waist-height ratio (*P* = 0.002), systolic blood pressure (*P* = 0.001), total cholesterol (*P* = 0.008), and triglyceride (*P* = 0.002) after adjusting for age and sex. With per-unit increase in TSH, the odds ratios of having abdominal obesity (OR = 1.18, 95% CI, 1.01–1.38) and triglyceride ≥ 150 mg/dL (OR = 1.18, 95% CI, 1.04–1.34) were significantly increased after adjusting for age, sex, and BMI.

**Conclusions:**

In adolescents with obesity, TSH was higher and FT4 was lower than in adolescents with normal weight. Hyperthyrotropinemia was associated with abnormal metabolic risk factors including abdominal obesity and elevated triglyceride.

## 1. Introduction

The prevalence of childhood obesity has significantly increased worldwide and has become an important global public health issue [1]. Childhood obesity is a major health concern, and endocrinopathies including thyroid dysfunction have been frequently reported. Obesity occurs when energy intake exceeds energy expenditure [2]. It is well established that thyroid hormone (TH) status correlates with body weight and energy expenditure [3, 4]. TH maintains basal metabolic rate, facilitates adaptive thermogenesis, modulates appetite and food intake, and regulates body weight [5].

Some cross-sectional studies have shown an association between high body mass index (BMI) and low levels of free T4 (FT4) or high levels of triiodothyronine (T3) and thyroid-stimulating hormone (TSH) within the euthyroid range [6, 7]. In adolescents with obesity, the most common thyroid abnormality is subclinical hyperthyrotropinemia [8–10]. Sometimes, subclinical hyperthyrotropinemia in subjects with obesity often may lead to a premature diagnosis of subclinical hypothyroidism and result in the initiation of inappropriate TH administration [11]. Reasons for hyperthyrotropinemia in adolescents with obesity remain unclear [12]. However, since alterations in levels of thyroid function test were often normalized with weight loss, they seem to be a reversible consequence of the weight status [10, 13]. The well-supported hypothesis explaining hyperthyrotropinemia in subjects with obesity is an adaptation process to increase energy expenditure for reducing the availability of energy for conversion into fat [12, 14].

Few reports are available regarding the relationship between obesity and thyroid function in Korean adolescents. Furthermore, there is still a lack of data regarding the relationship between thyroid function and other metabolic risk factors in children with obesity. In this study, we conducted a cross-sectional study based on data obtained in the 2013–2015 Korea National Health and Nutrition Examination Surveys (KNHANES) to investigate the status of thyroid function and its association with metabolic risk factors in Korean adolescents.

## 2. Materials and Methods

### 2.1. Data

Data was obtained from the KNHANES conducted between 2013 and 2015 by the Korean Ministry of Health and Welfare. The KNHANES are conducted annually using a rolling sampling design that involves a complex, stratified, multistage, and probability-cluster survey of a representative sample of the noninstitutionalized civilian population in South Korea. All individuals are randomly selected. Data was collected in a variety of ways, including household interviews, physical examinations, laboratory tests, and nutritional status assessments. The KNHANES data are publicly available with no charge [15].

### 2.2. Selection of the Study Population

**Figure 1:**
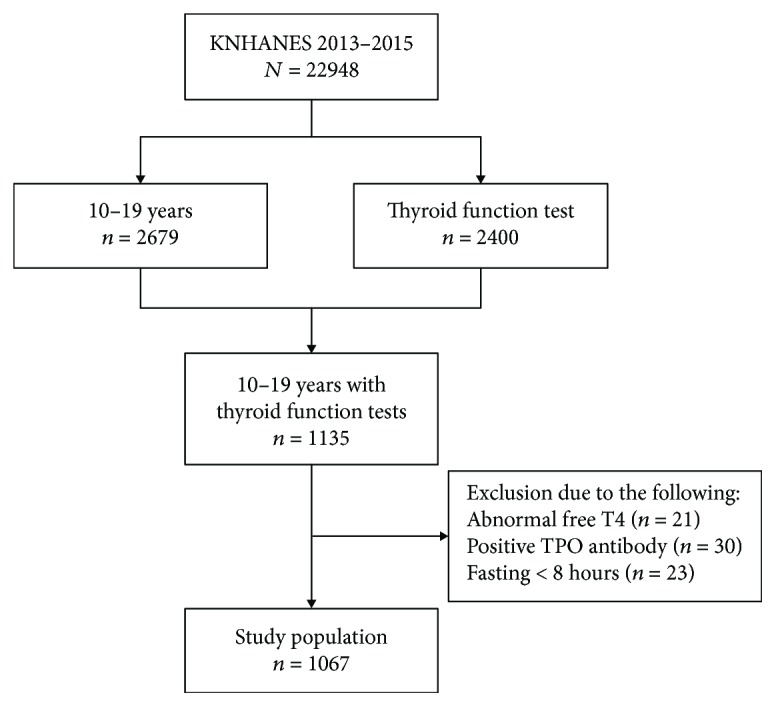
A flow chart for study population. KNHANES: Korea National Health and Nutrition Examination Survey; TPO: thyroid peroxidase.

In the 2013–2015 KNHANES, thyroid function test including FT4, TSH, and anti-thyroid peroxidase antibody (TPOAb) were performed on 2400 subjects aged ≥10 years old after subsampling of total study participants. Among 2679 subjects aged 10–19 years who participated in the 2013–2015 KNHANES, thyroid function tests were performed on 1135 subjects. Of these 1135 subjects, abnormal levels of FT4 (*n* = 21), positive TPOAb (*n* = 30), and inappropriate fasting states for laboratory measurements (*n* = 23) were excluded. Ultimately, our study population included 1067 adolescents (male = 559, female = 508) (Figure 1).

### 2.3. Anthropometric and Laboratory Measurements

Height was measured using a stadiometer (Seca225, Seca, Hamburg, Germany), and weight was measured with a balance beam scale (GL-6000-20, G-tech, Seoul, Korea) with participants wearing standard gowns. BMI was calculated as weight (kg) divided by height (m) squared. BMI data were calculated to *z*-score based on Korean reference data [16]. Waist circumference (WC) was measured to the nearest 0.1 cm at the end of normal expiration from the narrowest point between the midline of the most lateral border of the right and left iliac crest. Waist-height ratio (WHR) was calculated as WC (cm) divided by height (cm) and expressed as a percentage (%). Fasting plasma concentrations including glucose, total cholesterol (TC), triglyceride (TG), high-density lipoprotein-cholesterol (HDL-C), aspartate transaminase (AST), and alanine transaminase (ALT) were measured enzymatically using a Hitachi Automatic Analyzer 7600 (Hitachi, Tokyo, Japan) after subjects completed a minimum 8-hour overnight fast. Glycosylated hemoglobin (HbA1c) was measured using high-performance liquid chromatography (HLC-723G7; Tosoh, Tokyo, Japan), which is the method certified by the National Glycohemoglobin Standardization Program. Systolic blood pressure (SBP) and diastolic blood pressure (DBP) were measured using a mercury sphygmomanometer [Baumanometer Wall Unit 33(0850), W.A. Baum, New York, USA] according to the standardized protocol by trained personnel. An appropriately sized BP cuff was applied based on participants' arm circumference. BP was measured three times after sitting at least 5 minutes. The mean value of the last two readings was used for the analysis.

For analyzing the serum levels of TSH, FT4, and TPOAb, approximately 15 mL of blood was collected. After 30 minutes of separation of the serum, each sample was transferred to the testing facility and analyzed within 24 hours of collection. Serum TSH, FT4, and TPOAb levels were measured with an electrochemiluminescence immunoassay (Roche Diagnostics, Mannheim, Germany) after subjects completed a minimum 8-hour overnight fast. TSH was measured using an E-TSH kit (Roche Diagnostics), for which the reference range was 0.35 to 5.50 mIU/L. The FT4 was measured using an E-Free T4 kit (Roche Diagnostics), for which the reference range was 0.89 to 1.76 ng/mL. TPOAb was measured using an E-Anti-TPO kit (Roche Diagnostics); the normal range for TPOAb in humans is <34.0 IU/mL. The results for TSH, FT4, and TPOAb met specifications regarding accuracy, general chemistry, special immunology, and ligands established by the quality control and assurance program of the College of American Pathologists [17].

### 2.4. Study Criteria

Subclinical hyperthyrotropinemia was defined as elevated TSH (TSH > 5.5 IU/L) with normal FT4. A subject was classified as normal weight (below 85th percentile BMI for corresponding age and sex), overweight (85th ≤ BMI < 95th percentile for corresponding age and sex), or obese (BMI ≥ 95th percentile for corresponding age and sex) according to the 2017 Korea National Growth Charts [16]. Criteria for abnormal metabolic risk factors were adopted from those of components of metabolic syndrome defined by the International Diabetes Federation. WC ≥ 90th percentile for age and sex was used to diagnose abdominal obesity. Cut-off values of abnormal metabolic risk factors in adolescents were as follows: HDL-C < 40 mg/dL in boys and girls aged ≤15 years or <50 mg/dL in girls older than 16 years; TG ≥ 150 mg/dL; fasting glucose ≥ 100 mg/dL; SBP ≥ 130 mmHg or DBP ≥ 85 mmHg [18, 19].

### 2.5. Statistical Analyses

All statistical analyses were performed using Stata 14.2 software (StataCorp LP, College Station, TX, USA). Sampling weights were incorporated to produce valid population estimates that accounted for the complex survey design of the KNHANES. Data are presented as weighted mean ± standard error (SE) for continuous variables and frequency with weighted percentage for categorical variables. The anthropometric characteristics and laboratory data were compared between groups according to BMI category using linear regression analysis for continuous measures and logistic regression analysis for categorical measures. Multiple linear regression analysis was adjusted for age and sex to investigate relationships between TSH concentration and cardiometabolic risk factors. Multiple logistic regression models were used to calculate the adjusted odds ratio (OR) and 95% confidence interval (CI) for predicting the presence of metabolic risk factors by TSH concentrations after adjusting for potentially confounding variables. *P* values less than 0.05 were considered as statistically significant.

## 3. Results

### 3.1. Thyroid Function and Metabolic Risk Factors according to Increasing Levels of BMI Category in 10–19-Year-Old Korean Adolescents

**Figure 2:**
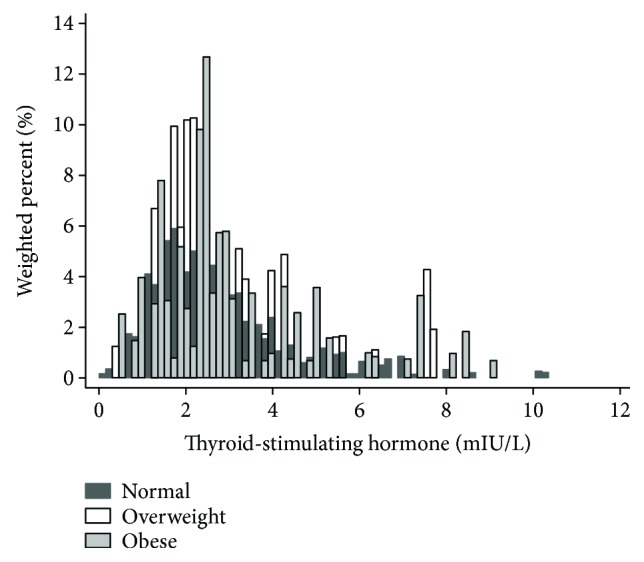
Distribution of thyroid-stimulating hormone by body mass index category.

The prevalence of Korean adolescents with normal weight, overweight, and obesity was 80.7% (*n* = 851), 8.7% (*n* = 103), and 10.6% (*n* = 113), respectively. With increasing levels of BMI category, the means of BMI (*P* for trend < 0.001), BMI *z*-score (*P* for trend < 0.001), WC (*P* for trend < 0.001), WHR (*P* for trend < 0.001), SBP (*P* for trend = 0.001), TC (*P* for trend = 0.004), TG (*P* for trend < 0.001), AST (*P* for trend = 0.043), ALT (*P* for trend < 0.001), and TSH (*P* for trend = 0.031) increased significantly (Table 1). With increasing levels of BMI category, the means of HDL-C (*P* for trend < 0.001) and FT4 (*P* for trend = 0.001) decreased (Table 1).  Figure 2 showed the distribution of TSH by BMI category. Among the three groups, there was no significant difference in the means of age, fasting glucose, HbA1c, and DBP (Table 1).

### 3.2. Linear Associations between Serum TSH Level and Metabolic Risk Factors in 10–19-Year-Old Korean Adolescents

Serum TSH level showed positive linear associations with BMI *z*-score (*β* = 0.09, *P* = 0.032), WHR (*β* = 2.92, *P* = 0.004), SBP (*β* = 0.015, *P* = 0.004), fasting glucose (*β* = 0.015, *P* = 0.002), TC (*β* = 0.81, *P* = 0.010), and TG (*β* = 0.30, *P* = 0.004), but no significant associations were observed with DBP, HbA1c, HDL-C, and ALT. After adjusting for age and sex, positive linear associations were persistently observed between serum TSH level and BMI *z*-score (*β* = 0.08, *P* = 0.031), WC (*β* = 0.014, *P* = 0.013), WHR (*β* = 2.92, *P* = 0.002), SBP (*β* = 0.015, *P* = 0.001), TC (*β* = 0.87, *P* = 0.008), and TG (*β* = 0.33, *P* = 0.002), but not with DBP, HbA1c, fasting glucose, and HDL-C (Table 2).

### 3.3. Multivariate Logistic Analyses of Having Metabolic Risk Factors according to Per-Unit Increase in TSH

In Korean adolescents, the odds ratios of having metabolic risk factors including abdominal obesity (OR = 1.22, 95% CI, 1.10–1.36), obesity (OR = 1.19, 95% CI, 1.05–1.35), and TG ≥ 150 mg/dL (OR = 1.21, 95% CI, 1.07–1.36) according to per-unit increase in TSH were significantly increased (model 1). The odds ratios of having abdominal obesity (OR = 1.18, 95% CI, 1.01–1.38) and TG ≥ 150 mg/dL (OR = 1.18, 95% CI, 1.04–1.34) according to per-unit increase in TSH were persistently observed after adjusting for age, sex, and BMI (model 3) (Table 3).

## 4. Discussions

In the present study, the serum TSH level in adolescents with obesity was higher than that in adolescents with normal weight. Suggested causes of subclinical hyperthyrotropinemia in a person with obesity are iodine deficiency, thyroid autoimmunity, mitochondrial dysfunction, thyroid hormone resistance, mutation in the TSH-receptor gene, impaired hypothalamic pituitary axis, or adaptation of increased energy expenditure mediated by leptin [12, 20–22]. However, hyperthyrotropinemia in obese subjects is usually not associated with iodine deficiency or autoimmune thyroiditis [10]. In our study, participants with positive TPOAb were excluded. Furthermore, mutations in the TSH-receptor gene are very rare [23]. Overall, the most favored hypothesis attempting to explain the hyperthyrotropinemia in subjects with obesity is the increased leptin-mediated production of pro-thyrotropin-releasing hormone (TRH) [14, 23].

Obesity is associated with serum leptin levels [24]. In addition to TRH/TSH regulation by TH feedback, there is central modulation by nutritional signals. Leptin is a known regulator of TRH and TSH secretion via direct action on the paraventricular nucleus and indirect action on the arcuate nucleus [25]. In the hypothalamus, leptin might increase thermogenesis by regulating TRH neurons [26]. Mantzoros et al. showed that leptin and plasma TSH levels are both highly organized and pulsatile, with similar circadian rhythms, and using a cosinor analysis, they showed near-superimposable peak values [27]. On the other hand, some reports suggested that TSH stimulates leptin secretion via a direct effect on adipocytes [28]. These complex and dual relationships between leptin and the thyroid axis left open the possibility that hyperthyrotropinemia in obese subjects may be affected by regulation of leptin pulses.

In the present study, the serum FT4 level in adolescents with obesity was lower than that in the normal weight group. Previous studies have also reported the decreased serum FT4 level within the normal range in obese subjects [29]. The involved mechanisms are not clear although these alterations have been explained by increased deiodinase activity in obesity. Local conversion of thyroxine (T4) to T3 by 5′-deiodinase type 2 (D2) is a key mechanism of TH regulation [5]. The high conversion rate of T4 to T3 in subjects with obesity has been also interpreted as a defense mechanism, capable of counteracting the accumulation of fat by increasing the basal metabolic rate, positively related to the levels of total T3 and free T3 [30, 31]. Leptin has been also shown to regulate D2 in different tissues, depending on energetic status, thus promoting the conversion of T4 to T3 [21, 32].

We found that the ORs of having abdominal obesity, obesity, and hyper TG were increased according to the per-unit increase in TSH in Korean adolescents. Some reports suggest the correlations between hyperthyrotropinemia and TG, insulin resistance, and DBP in obese adolescents [25, 33]. Emerging evidence identifies a role for TH in metabolism of lipids, carbohydrates, proteins, and heat production [12]. Some suggest that the associations between hyperthyrotropinemia and lipid profiles in children with obesity are secondary to weight status rather than thyroid dysfunction [34]. However, in our study, correlations between serum levels of TSH and TG in multiple regression analysis still persist significantly after adjusting BMI. This significant association between hyperthyrotropinemia with TG might support the role for TH in metabolism of lipids.

There are some limitations in this study. We did not have data on serum T3 and leptin levels. Because of the cross-sectional design of this study, long-term consequences of hyperthyrotropinemia in obese adolescents could not be elucidated. However, to the best of our knowledge, this is the first study to show the status of thyroid function and the association with metabolic risk factors in Korean adolescents with obesity, which was based on the reliable large-scale nationally representative dataset.

In conclusion, a higher serum TSH and lower FT4 level was observed in Korean adolescents with obesity than in those with normal weight. Positive linear associations were observed between serum TSH levels and BMI-SDS, WC, WHR, SBP, TC, and TG after adjusting for age and sex. We also found increased odds ratios of having abdominal obesity, hyper TG, and obesity according to the per-unit increase in TSH after adjusting for age, sex, and BMI. Further investigation focusing on the hypothalamus-pituitary-thyroid axis and metabolic risk factors in obese children including energy metabolism with a large data set is necessary.

## Figures and Tables

**Table 1:** Characteristic and laboratory data according to obesity, sex, and age in 10–19-year-old Korean adolescents.

	Normal	Overweight	Obesity	*P* for trend
*N* (%)	851 (80.7%)	103 (8.7%)	113 (10.6%)	—
Estimated number of subjects	2,113,591	228,910	276,292	—
Sex (male, %)	443 (51.0%)	57 (53.7%)	59 (53.0%)	0.859
Age (yr)	15.2 ± 0.1	14.5 ± 0.3	14.9 ± 0.2	0.468
BMI (kg/m^2^)	20.2 ± 0.1	24.5 ± 0.1	28.7 ± 0.3	<0.001
BMI *z*-score	−0.29 ± 0.03	1.32 ± 0.02	2.54 ± 0.08	<0.001
WC (cm)	69.0 ± 0.3	79.1 ± 0.6	88.4 ± 1.1	<0.001
WHR (%)	42.5 ± 0.2	48.3 ± 0.3	53.5 ± 0.5	<0.001
Systolic BP (mmHg)	107.8 ± 0.4	112.6 ± 1.1	115.6 ± 1.0	<0.001
Diastolic BP (mmHg)	66.7 ± 0.3	67.4 ± 1.1	68.0 ± 0.8	0.133
Fasting glucose (mg/dL)	90.7 ± 0.3	91.7 ± 0.7	94.8 ± 2.5	0.081
HbA1c (%)	5.40 ± 0.01	5.45 ± 0.04	5.59 ± 0.11	0.064
Total cholesterol (mg/dL)	156.9 ± 0.9	159.2 ± 3.3	165.5 ± 2.8	0.004
HDL-C (mg/dL)	52.2 ± 0.4	49.2 ± 1.1	45.0 ± 0.9	<0.001
Triglyceride (mg/dL)	72.4 ± 1.3	84.4 ± 5.1	90.6 ± 4.6	<0.001
AST (IU/L)	18.4 ± 0.3	18.8 ± 0.9	19.9 ± 0.6	0.043
ALT (IU/L)	14.0 ± 0.5	17.5 ± 2.1	23.7 ± 1.5	<0.001
Free T4 (ng/mL)	1.30 ± 0.01	1.26 ± 0.02	1.25 ± 0.02	0.001
TSH (mIU/L)	2.73 ± 0.06	2.77 ± 0.02	3.24 ± 0.22	0.031
Subclinical hyperthyrotropinemia, *n* (%)	54 (5.8%)	6 (4.9%)	11 (10.0%)	0.221
Abdominal obesity, *n* (%)	17 (2.3%)	19 (19.4%)	86 (77.6%)	<0.001
Hypertension, *n* (%)	24 (2.4%)	6 (9.2%)	12 (10.6%)	<0.001
Hyperglycemia, *n* (%)	74 (8.4%)	13 (10.6%)	14 (13.3%)	0.250
Hypertriglyceridemia, *n* (%)	47 (5.5%)	18 (15.8%)	19 (16.2%)	<0.001
Hypo-HDL-C, *n* (%)	96 (12.1%)	16 (14.0%)	31 (26.3%)	<0.001
Metabolic syndrome, *n* (%)	4 (0.4%)	2 (1.9%)	15 (13.9%)	<0.001

Data are presented as weighted mean ± standard error or frequency (weighted percentage). TC, HDL-C, and TG were log-transformed for analysis and expressed as geometric mean ± standard error. BMI: body mass index; WC: waist circumference; WHR: waist-height ratio; BP: blood pressure; HDL-C: high-density lipoprotein cholesterol; AST: aspartate transaminase; ALT: alanine transaminase; TSH: thyroid-stimulating hormone.

**Table 2:** Results of linear regression analysis of factors associated with TSH concentration.

Variables	Unadjusted			Adjusted for sex and age		
	Coefficient *β*	S.E.	*P* value	Coefficient *β*	S.E.	*P* value
BMI *z*-score	0.09	0.04	0.032	0.08	0.04	0.031
Waist circumference	0.008	0.005	0.118	0.014	0.006	0.013
Waist-height ratio	2.92	1.01	0.004	2.90	0.98	0.002
Systolic BP	0.015	0.005	0.004	0.017	0.005	0.001
Diastolic BP	0.003	0.007	0.666	0.011	0.007	0.103
HbA1c	0.27	0.14	0.051	0.21	0.15	0.170
Fasting glucose	0.015	0.004	0.002	0.011	0.006	0.052
Total cholesterol	0.81	0.31	0.010	0.87	0.33	0.008
Triglyceride	0.30	0.10	0.004	0.33	0.10	0.002
HDL-C	0.18	0.29	0.535	0.21	0.29	0.464
ALT	0.0001	0.004	0.965	0.001	0.004	0.821

TC, TG, and HDL-C were log-transformed for analysis. TSH: thyroid-stimulating hormone; BMI: body mass index; BP: blood pressure; HbA1c: glycated hemoglobin; HDL-C: high-density lipoprotein cholesterol; ALT: alanine transaminase.

**Table 3:** Multivariate logistic analyses of predicting obesity and metabolic risk factors according to per-unit change in TSH concentration.

	Model 1		Model 2		Model 3	
	OR (95% CI)	*P* value	OR (95% CI)	*P* value	OR (95% CI)	*P* value
Abdominal obesity	1.22 (1.10, 1.36)	<0.001	1.22 (1.10, 1.36)	<0.001	1.18 (1.01, 1.38)	0.034
Obesity	1.19 (1.05, 1.35)	0.007	1.19 (1.05, 1.35)	0.008	1.08 (0.91, 1.28)	0.408
Hypertension	1.11 (0.94, 1.32)	0.226	1.14 (0.96, 1.35)	0.142	1.10 (0.91, 1.32)	0.326
Hyperglycemia	1.07 (0.96, 1.20)	0.235	1.03 (0.92, 1.16)	0.573	1.02 (0.91, 1.15)	0.717
Elevated triglyceride	1.21 (1.07, 1.36)	0.002	1.20 (1.06, 1.36)	0.003	1.18 (1.04, 1.34)	0.011
Decreased HDL-C	1.03 (0.92, 1.15)	0.625	1.08 (0.96, 1.21)	0.187	1.06 (0.94, 1.19)	0.321
Metabolic syndrome	1.17 (0.96, 1.42)	0.128	1.19 (0.99, 1.43)	0.070	1.13 (0.87, 1.47)	0.345

Model 1: unadjusted; Model 2: adjusted for age and sex; Model 3: adjusted for age, sex, and body mass index; HDL-C: high-density lipoprotein cholesterol.

## Data Availability

The raw data used to support the findings of this study are available at the KNHANES webpage (https://knhanes.cdc.go.kr/knhanes/eng/index.do). Requests for access to these data could be directed to the officer at the Korea Centers for Disease Control and Prevention (sun4070@korea.kr; +82-43-719-7467).
